# Astragaloside-IV prevents acute kidney injury and inflammation by normalizing muscular mitochondrial function associated with a nitric oxide protective mechanism in crush syndrome rats

**DOI:** 10.1186/s13613-017-0313-2

**Published:** 2017-09-04

**Authors:** Isamu Murata, Yuji Abe, Yuka Yaginuma, Kayako Yodo, Yuka Kamakari, Yurika Miyazaki, Daichi Baba, Yuko Shinoda, Toru Iwasaki, Kunihiko Takahashi, Jun Kobayashi, Yutaka Inoue, Ikuo Kanamoto

**Affiliations:** 1 Laboratory of Drug Safety Management, Faculty of Pharmacy and Pharmaceutical Science, Josai University, Keyakidai 1-1, Sakado, Saitama 350-0295 Japan; 2 Water and Food Inspection Group, Saitama Prefectural Institute of Public Health, Saitama, Japan; 3 Hygiene Inspection Section, Koshigaya City Public Health Center, Saitama, Japan; 4 Division of Pathophysiology, Department of Clinical Dietetics and Human Nutrition, Faculty of Pharmaceutical Science, Josai University, Saitama, Japan

**Keywords:** Crush syndrome, Astragaloside-IV, Nitric oxide, Fluid resuscitation, Acute kidney injury, Anti-inflammatory, Mitochondria dysfunction

## Abstract

**Background:**

Crush syndrome (CS) is a serious medical condition characterized by muscle cell damage resulting from decompression after compression (i.e., ischemia/reperfusion injury). A large number of CS patients develop cardiac failure, kidney dysfunction, and systemic inflammation, even when fluid therapy is administered. We evaluated whether the administration of astragaloside-IV (AS)-containing fluid improved survival by preventing kidney and muscular mitochondrial dysfunction in a rat model of CS.

**Results:**

The CS model was generated by subjecting anesthetized rats to bilateral hind limb compression with a rubber tourniquet for 5 h. Rats were then randomly divided into four groups: (1) sham; (2) CS with no treatment; (3) CS with normal saline treatment; and (4) CS with normal saline + 10 mg/kg AS. AS-containing fluid improved kidney function by improving shock and metabolic acidosis in CS rats. In addition, there was a reduction in oxidative damage. The attenuation of hyperkalemia was significantly related to improving muscle injury via preventing mitochondrial dysfunction. Moreover, this mitochondria protection mechanism was related to the nitric oxide (NO) generated by activation of endothelial nitric oxide synthase, which provided an anti-oxidative and anti-inflammatory effect.

**Conclusions:**

Treatment with AS-containing fluid led to a dramatic improvement in survival following CS because of direct and indirect anti-oxidative effects in the kidney, and improvements in mitochondrial dysfunction and inflammation owing to AS acting as an NO donor in injured muscle.

**Electronic supplementary material:**

The online version of this article (doi:10.1186/s13613-017-0313-2) contains supplementary material, which is available to authorized users.

## Background

Crush syndrome (CS) is a serious medical condition characterized by circulatory shock, acute kidney injury (AKI), and systemic inflammation that occurs after traumatic events such as earthquakes, landslides, and traffic accidents [1]. It is associated with a high mortality rate, especially in the acute phase. CS develops after decompression of limb muscles (reperfusion) following prolonged compression (ischemia) of a large portion of skeletal muscle, resulting in rhabdomyolysis (muscle cell breakdown). However, in addition to its local effects, CS can cause systemic failure resulting from acute respiratory distress syndrome (ARDS), systemic inflammatory response syndrome (SIRS), multiple organ failure, and confounding late-phase symptoms [2].

Fluid therapy is the first-line treatment for CS. Shock and acute kidney failure can be prevented by early infusion of fluid. Electrolyte abnormalities are common in patients with crush-related acute kidney failure, and fatal hyperkalemia is the most severe abnormality, for which treatment with normal saline containing sodium bicarbonate is recommended [3]. In the clinical setting, fluid resuscitation therapy is beneficial for early symptoms; however, it is difficult to prevent an inflammatory condition from developing after an ischemia/reperfusion injury (IRI).

Astragaloside-IV (3-*O*-beta-d-xylopyranosyl-6-*O*-beta-d-glucopyranosyl-cycloastragenol, AS), isolated from *Astragalus membranaceus*, is a bioactive saponin of astragalosides. Accumulating evidence has demonstrated the anti-inflammatory effects of AS against mitochondrial damage [4, 5] and its anti-oxidative effects [6, 7]. In addition, AS has been shown to protect against acute kidney injury [8] and kidney tubular injury [9], which is important for CS therapy. These effects of AS are believed to involve nitric oxide (NO), because they are consistent with previously demonstrated benefits of NO generation via endothelial nitric oxide synthase (eNOS). This mechanism early after decompression prevents muscle damage induced by ischemia/reperfusion via the protective effects of NO and the suppression of systemic inflammation, thereby increasing survival rates in CS [10, 11]. AS therapy has been proposed as an early intervention to treat CS in the clinic. However, few studies have focused on these mechanisms of AS. Therefore, we hypothesize that treatment with AS inhibits mitochondrial dysfunction via an eNOS activation mechanism in injured muscle in CS model rats, and the experiments presented herein are designed to test that hypothesis.

## Methods

### Animal model of CS

Male Wistar rats weighing 250–300 g were obtained from Japan SLC (Shizuoka, Japan) and housed in a room maintained at a temperature of 23 ± 3 °C and a relative humidity of 55 ± 15% under a 12:12-h light/dark cycle, with free access to food and water. Animal experiments were carried out according to the guidelines for animal use and approved by the Life Science Research Center of Josai University (approval nos. H26027, H26028, H27025, and H27027). Anesthesia was induced by intraperitoneal injection of sodium pentobarbital (50 mg/kg body weight). Body temperature was maintained throughout the experiment using a heating pad. The CS model was established as previously reported [12]. Briefly, a rubber tourniquet was applied to the bilateral hind limb of each rat, wrapped five times around a 2.0 kg metal cylinder, and the end of the band was glued. After a compression of 5 h, the compression was released by cutting the band and removing the tourniquet.

### AS preparation

The chemical structure of AS (Carbosynth, Compton, UK) is depicted in Additional file 1: Fig. S1. The probable purity of AS was 99.6 ± 0.8%, as determined using the partial modification HPLC method (HPLC conditions: mobile phase: 50% acetonitrile, flow rate: 1.0 mL/min, column: Inertsil-ODS3 (4.6 mm × 250 mm, *φ* 5 μm), column temperature: 40 °C, detector wavelength: 203 nm) [13]. A 1,1-diphenyl-2-picrylhydrazyl (DPPH) antioxidant assay of AS was performed as Sharma et al. [14] previously reported. Ascorbic acid (AA) has a high antioxidant capacity and was used as an antioxidant reference for comparison with AS.

### Experimental design

- *Experimental-1 (AS dosage study)* The AS dosages were selected using previous reports by Li et al. [15] to determine the effective dosage in CS rats. To determine the optimal dose of AS, animals were randomly divided into seven groups: (1) sham; (2) CS with no treatment (CS-only group); and (3–7) CS with treatment at 1, 2, 5, 10, and 20 mg/kg AS (named C-1, 2, 5, 10, and 20 AS-one group, respectively) via tail vein bolus injection at decompression just before from a rubber tourniquet.
- *Experimental-2 (therapy effect study)* To examine the effects of AS in CS, animals were randomly divided into four groups: (8) sham; (9) CS with no treatment (CS-only group); (10) CS with normal saline treatment (C-saline group); and (11) CS with normal saline + 10 mg/kg AS (C-AS group). The C-saline and C-AS groups were subjected to decompression with the rubber tourniquet, immediately followed by 3 h of reperfusion by massive fluid resuscitation at a rate of 30 mL/(kg h^−1^).
- *Experimental-3 (pharmacokinetics (PK) study)* We analyzed the blood concentration profile and PK parameters of AS administered by tail vein to sham and CS rats. Animals were randomly assigned to four groups: sham, sham-AS, CS-only, and C-AS groups.

### Analysis of mean arterial pressure (MAP), blood gas levels, biochemical parameters, coagulation, and NO and cytokine levels

MAP was recorded using a PowerLab data acquisition system (AD Instruments, Nagoya, Japan). One carotid artery was cannulated with a polyethylene catheter (PE-50 tubing) connected to a pressure transducer. Arterial blood samples from each group were obtained 1, 3, 6, and 24 h after reperfusion using a carotid artery catheter. The pH, partial pressure of oxygen (*Pa*O_2_), partial pressure of carbon dioxide (*Pa*CO_2_), bicarbonate (HCO_3_^−^) concentration, and base excess of arterial blood were analyzed using an i-STAT300F blood gas analyzer (FUSO Pharmaceutical Industries, Osaka, Japan).

In each experimental group (0, 1, 3, 6, and 24 h after reperfusion, *n* = 3–6), venous blood and tissue samples from the gastrocnemius muscles and kidneys were assayed for thiobarbituric acid reactive substances (TBARS), myeloperoxidase (MPO) activity, skeletal muscle edema index, mitochondrial permeability transition (MPT), superoxide dismutase (SOD) activity, Western blotting, and histology at each time point. For histology, tissues were fixed in 10% formalin and embedded in paraffin wax. Sections were cut and stained with hematoxylin and eosin and then carefully examined microscopically. Venous blood samples from each group were obtained using a jugular catheter. Venous blood from the jugular vein was collected and centrifuged to measure plasma levels of potassium (K^+^), blood urea nitrogen (BUN), creatinine (Cre), and creatine phosphokinase (CPK) (measurements were carried out by SRL Inc., Tokyo, Japan). Red blood cells (RBC), white blood cells (WBC), and platelets were measured using a Celltac hematology analyzer (Nihon Kohden Co., Tokyo, Japan). The nitrite (NO_2_^−^) and nitrate (NO_3_^−^) concentrations in muscle and plasma were measured using a dedicated HPLC system (ENO-20, Eicom, Kyoto, Japan), according to a previously reported method [16]. Interleukin-1 beta (IL-1β) and tumor necrosis factor alpha (TNF-α) were measured by ELISA according to the manufacturer’s instructions (Rat IL-1β/IL-1F2 Quantikine ELISA Kit and Rat TNF-α Quantikine ELISA Kit, R&D Systems, Inc., MN, USA).

### Assessment of kidney function

The bladder was cannulated with PE-50 tubing concomitantly with the jugular vein. Urine samples were obtained starting 1 h prior to decompression until immediately after decompression (0 h). Urine samples were then collected every 1 h for 24 h and centrifuged at 1500×*g* for 5 min at 20–25 °C. Kidney function was determined based on glomerular filtration rate (GFR), urine volume, urine osmotic pressure (Osmomat 030-D; Gonotec GmbH, Berlin, Germany), *N*-acetyl-β-d-glucosaminidase (NAG) levels (Shionogi & Co., Osaka, Japan), and urine pH (Pretest 5bII; Wako Pure Chemical Industries, Tokyo, Japan). Kidney injury marker-1 (KIM-1) was measured by ELISA according to the manufacturer’s instructions (Rat TIM-1/KIM-1/HAVCR Immunoassay, R&D Systems, Inc.).

### Determination of reactive oxygen species (ROS) production, MPO activity, and mitochondrial function

ROS production in the injured gastrocnemius muscle was determined by measuring the concentration of TBARS. MPO activity in the blood and muscle tissue was measured as previously described [10]. The relative weight (g/100 g body weight) of the injured muscle (gastrocnemius muscle) was determined, which acted as an index of skeletal muscle edema in the affected limbs. Isolation of mitochondria for evaluation of mitochondrial function in crush injury muscle was performed using a mitochondrial isolation kit for tissue (Thermo Fisher Scientific K.K., Kanagawa, Japan). In the MPT, mitochondrial membrane potential (i.e., mitochondrial inner membrane function) was evaluated using a JC-1 mitochondrial membrane potential assay kit (Cayman Chemical Company, Ann Arbor, MI, USA). To evaluate mitochondrial outer membrane function, cytochrome *c* (cyt *c*) of crush injured muscle cytoplasm (in the samples that did not include mitochondria) was quantified using a Quantikine^®^ cyt *c* Immunoassay (R&D Systems, Inc.). SOD activity was determined using a SOD Assay Kit—WST (Dojindo Laboratories, Tokyo, Japan). All operation procedures were performed in accordance with the corresponding instruction manuals.

### Western blotting

Western blotting was carried out as previously described [10]. Briefly, rat muscle and kidney tissue were homogenized and centrifuged, and proteins in the lysate were separated by sodium dodecyl sulfate–polyacrylamide gel electrophoresis. Proteins were detected on membranes using antibodies against eNOS (Cell Signaling Technology, Tokyo, Japan), phospho-eNOS (Ser1177) (p-eNOS; Cell Signaling Technology), inducible NO synthase (iNOS; Cell Signaling Technology), heme oxygenase (HO)-1 (Thermo Fisher Scientific K.K.), α-tubulin (Cell Signaling Technology), and hypoxia-inducible factor-1 alpha (HIF-1α) (Abcam PLC, Tokyo, Japan). Protein bands were visualized using an enhanced chemiluminescence detection system (SuperSignal West Dura Extended Duration Substrate; Pierce Biotechnology, Tokyo, Japan) with horseradish peroxidase-conjugated secondary antibodies (Pierce Biotechnology). Band intensities were quantified using a ChemiDoc XRS + Molecular Imager with Image Lab software (Bio-Rad Laboratories, Hercules, CA, USA), with α-tubulin used as a loading control.

### Sampling and sample preparation for PK parameters

Blood samples from each group were obtained 0 (just before administration), 0.083, 0.25, 0.5, 1, 1.5, 2, 2.5, 3, 3.5, 4, 4.5, 5, 5.5, 6, 7, 8, 9, and 24 h after reperfusion via a jugular catheter. Sample preparations were optimized as follows: 150 µL of plasma and urine samples were extracted with 150 µL methanol. These samples were vortexed and centrifuged at 12,000×*g* for 20 min at 4 °C. Two hundred microliters of the supernatant was mixed with 790 μL water and 10 μL 0.05 µg/mL digoxin (DIG, internal standard (IS), Wako Pure Chemical Industries, Ltd.), and these samples were subjected to cleanup on an active Strata™ X solid phase extraction column (Shimadzu GLC Ltd., Tokyo, Japan).

### Instrumental and conditions for PK parameters of AS

AS concentrations were measured using LC–MS/MS methods with an ACQUITY UPLC TQD (Waters, Milford, MA, USA). Masslynx 4.1 software was used for instrumental control, and acquisition and processing of the data. UPLC separation was performed using an ACQUITY UPLC BEH C18 1.7 µm 2.1 × 100 mm column (Waters) kept at 40 °C. An MS detector with an electrospray ionization (ESI) interface in negative ion mode (ESI^−^) was used for quantitative analysis, with acquisition in multiple reaction monitoring (MRM) mode. The *m*/*z* ratios were as follows: *m*/*z* 843.5[*M* + CH3COO^−^] > 843.5 for AS, *m*/*z* 549.4[*M* + CH_3_COO^−^] > 549.3 for cycloastragenol (CAG), and 839.5[*M* + CH3COO^−^] > 839.5 DIG. The following linear gradient program was applied to the analyte, using a mobile phase consisting of deionized water containing 0.1% acetic acid and 10 mM ammonium acetate (A solution) and acetonitrile (B solution): 0–2 min, 60:40–10:90; 2–7 min, 10:90; and 7–12 min, 60:40 (A:B v/v). The sample injection volume was 5 μL. The flow rate was maintained at 0.4 mL/min. The instrumental parameters of the MS detector were optimized as follows: capillary voltage, 3000 V; cone voltage, 30 and 50 V (50 V; AS and 30 V; DIG and CAG); extractor voltage, 3 V; RF lens voltage, 0.2 V; source temperature, 120 °C; desolvation temperature, 350 °C; cone gas flow, 50 L/h; desolvation gas flow, 600 L/h; collision gas flow, 0.15 mL/min; collision energy, 5 eV. The linear calibration curves were plotted between 0.01, 0.05, 0.1, 0.5, 1, 5, and 10 µg/mL (*R* ^2^ > 0.999). The minimum limit of quantifiable concentration was less than 0.01 µg/mL. The following PK parameters were calculated: area under the curve (AUC), mean residence time (MRT), total body clearance (CL_tot_), steady-state volume of distribution (*V*_ss_), elimination rate constant (*K*_el_), biological half-life (*T*_1/2_), maximum drug concentration time (*T*_max_), and maximum blood concentration (*C*_max_) using standard procedures.

### Statistical analysis

Results are expressed as mean ± SEM. Differences between groups were assessed by analysis of variance with Tukey’s honest significant difference test or Tukey’s test. Survival curves were generated by the Kaplan–Meier method, and survival was compared by the log-rank test. The PK parameters and blood profiles of AS were compared by the Student’s *t* test. Differences were considered significant for *p* values <0.05.

## Results

### Optimizing AS dose on the basis of rat survival following bolus injection

The survival rate of the CS-only group was 0% (0/10) 48 h after reperfusion. The survival rates of the C-1, 2, 5, 10, and 20 AS-one groups were 0% (0/10), 0% (0/10), 0% (0/10), 40% (4/10), and 0% (0/10) 48 h after reperfusion. The increase in mortality in the C-20 AS-one group compared to that in the C-10 AS-one group was caused by the development of significant metabolic and respiratory alkalosis in the former group (Additional file 2: Table S1). Moreover, the mean blood pressure (MBP) of the C-10 AS-one group was more stabilized than that of the CS-only group 1 and 3 h after reperfusion (Additional file 3: Fig. S2). Therefore, we used 10 mg/kg AS-containing saline in CS rats in further studies.

### AS treatment improves hyperkalemia, shock, and metabolic acidosis

**Fig. 1:**
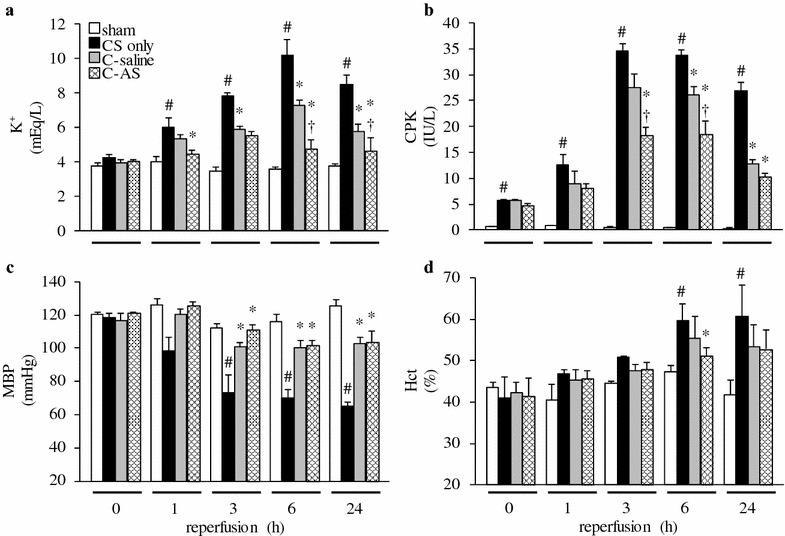
Effect of fluid resuscitation on K^+^, CPK, MBP, and Hct levels in CS rats. **a** K^+^ concentration, **b** CPK concentration, **c** MBP, and **d** Hct level. *White bar* sham, *black bar* CS-only, *gray bar* C-saline, and *shaded bar* C-AS. Values represent mean ± SEM (*n* = 3). ^#^ *p* < 0.05 versus sham group, *p < 0.05 versus CS-only group, and ^†^ *p* < 0.05 versus C-saline group (Tukey’s test). *CPK* creatine phosphokinase, *MBP* mean blood pressure, *Hct* hematocrit, *CS* crush syndrome

K^+^, CPK, and hematocrit (Hct) in the CS-only group were significantly increased compared to those of the sham group. In contrast, MBP in the CS-only group was significantly decreased compared to that in the sham group (Fig. 1). HR, SBP, and DBP in the CS-only group were significantly decreased compared to those in the sham group (Additional file 4: Table S2). Injured muscle edema in the CS-only group was significantly increased compared to that in the sham group during the experimental period. In contrast, the fluid resuscitation (i.e., C-saline and C-AS) groups had a tendency toward increased muscle edema compared to the CS-only group (Additional file 5: Fig. S3 and Additional file 6: Table S3). The blood gas parameters in the CS-only group indicated severe metabolic acidosis compared to those in the sham group (Additional files 7 and 8: Tables S4 and S5). By contrast, these changes in the fluid administration groups (i.e., C-saline and C-AS groups) were significantly improved over those in the CS-only group. Moreover, K^+^ and CPK levels and blood gas parameters in the C-AS group were significantly improved compared to those in the C-saline group.

In addition, no adverse effects (i.e., hemolytic action) were observed following AS treatment. These would be characterized by saponin stimulation; however, hemoglobin (Hb), mean corpuscular volume (MCV), mean corpuscular hemoglobin (MCH), and mean corpuscular hemoglobin concentration (MCHC) levels were not significantly different among the experimental groups (data not shown).

### AS treatment improves kidney function in CS

**Fig. 2:**
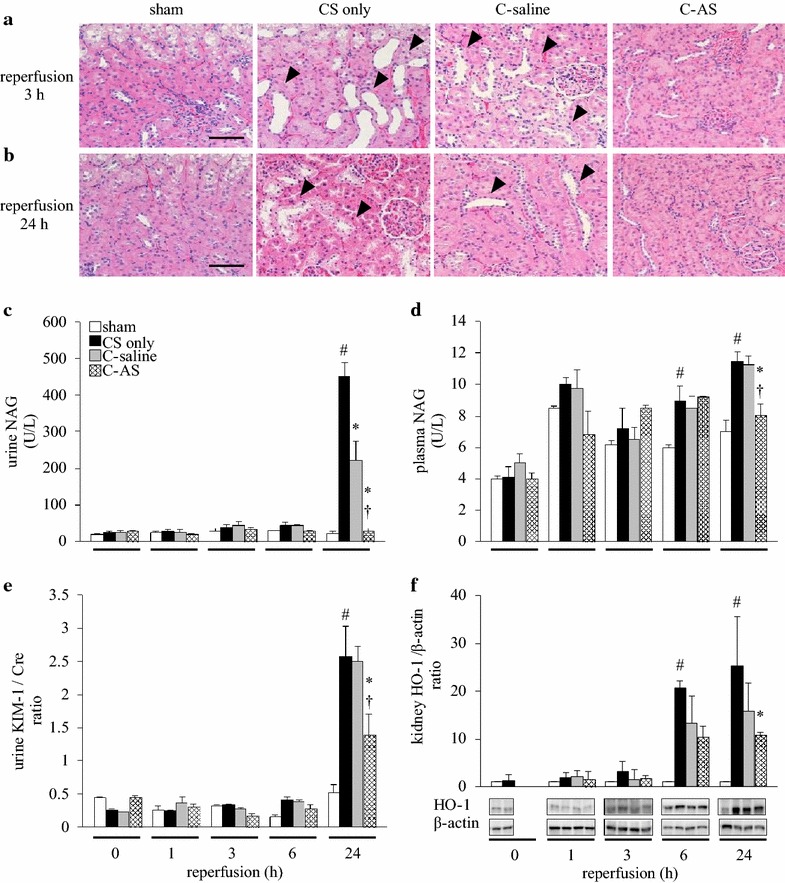
Effect of fluid resuscitation on kidney function in CS rats. **a** Hematoxylin and eosin-stained kidney sections following reperfusion for 3 h, **b** hematoxylin and eosin-stained kidney sections following reperfusion for 24 h, **c** urine NAG concentration, **d** plasma NAG concentration, **e** urine KIM-1/Cre ratio, and **f** kidney HO-1/β-actin ratio. *White bar* sham, *black bar* CS-only, *gray bar* C-saline, and *shaded bar* C-AS. Values represent mean ± SEM (*n* = 3). ^#^ *p* < 0.05 versus sham group, *p < 0.05 versus CS-only group; and ^†^ *p* < 0.05 versus C-saline group (Tukey’s test). Micrographs are representative of three independent experiments (×200 magnification; *scale bars* 100 μm). *Black arrowhead* dilated kidney tubule. *CS* crush syndrome, *NAG N*-acetyl-β-d-glucosaminidase, *KIM-1* kidney injury marker-1, *Cre* creatinine, *HO-1* heme oxygenase-1

Parameters of kidney function (i.e., BUN, Cre, pH, osmotic pressure, urine volume, and GFR levels) are shown in Additional files 9 and 10: Tables S6 and S7. Acute kidney injury in CS is characterized by acute tubular necrosis, formation of myoglobin casts, and dilation of distal convoluted tubules, followed by myoglobinuric nephropathy. Therefore, we evaluated kidney function by focusing on the failure of distal convoluted tubules. Pathological changes were not observed in the sham group. The CS-only group displayed moderate dilatation and injury to the tubules after 3 and 24 h of reperfusion (Fig. 2a, b). Urine NAG, plasma NAG, and KIM-1/Cre ratios in the CS-only group were not significantly different from those in the sham group from 0 to 6 h after reperfusion, but were significantly increased after 24 h of reperfusion (Fig. 2c–e). Moreover, HO-1 expression levels in the CS-only group were significantly increased compared to those in the sham group 6 and 24 h after reperfusion (Fig. 2f). By contrast, these changes in the CS-only and C-saline groups were significantly improved in the C-AS group. In addition, urine pH in the C-AS group was significantly improved compared to that in the CS-only and C-saline groups (Additional files 9 and 10: Tables S6 and S7).

### Vascular endothelial damage is attenuated by AS-containing fluid resuscitation via anti-inflammatory effects

**Fig. 3:**
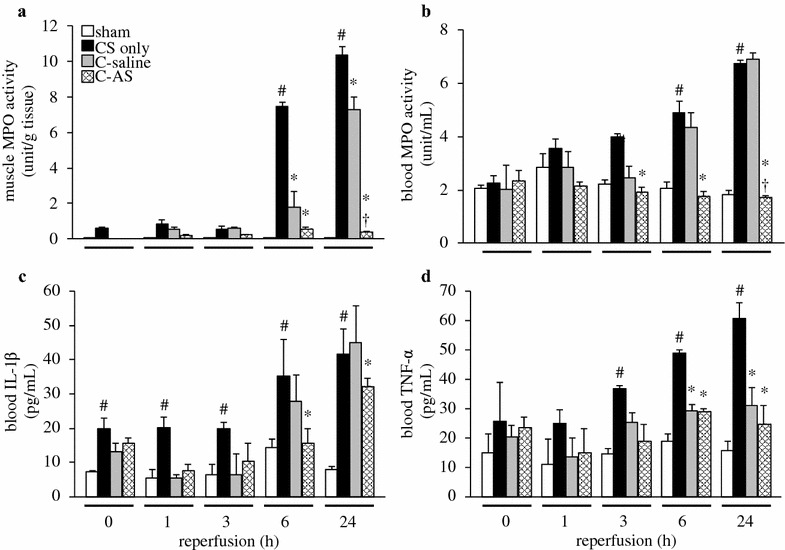
Effect of fluid resuscitation on inflammatory mediators in CS rats. **a** Muscle MPO activity, **b** blood MPO activity, **c** blood IL-1β, and **d** blood TNF-α. *White bar* sham, *black bar* CS-only, *gray bar* C-saline, and *shaded bar* C-AS. Values represent mean ± SEM (*n* = 3). ^#^ *p* < 0.05 versus sham group, *p < 0.05 versus CS-only group, and ^†^ *p* < 0.05 versus C-saline group (Tukey’s test). *CS* crush syndrome, *MPO* myeloperoxidase, *IL-1β* interleukin-1 beta, *TNF-α* tumor necrosis factor-α

Figure 3 indicates that leukocyte activity and filtration improved owing to decreased IL-1β and TNF-α levels. Muscle MPO activity in the CS-only group was significantly increased compared to that in the sham group at 6 and 24 h. Moreover, blood MPO activity, IL-1β, and TNF-α expression were similarly increased at 3, 6, and 24 h. By contrast, these changes in the C-AS group were significantly improved compared to those in the CS-only and C-saline groups. In addition, WBC levels were higher in the CS-only, C-saline, and C-AS groups after 3, 6, and 24 h of reperfusion than those in the sham group were. The platelet level in the CS-only group was significantly lower than that in the sham group. In contrast, the WBC level in the C-AS group tended to be lower than that in the CS-only group, and platelet levels were not significantly increased compared to those in the CS-only group (Additional file 11: Table S8).

### Muscle tissue injury is attenuated by avoidance of mitochondrial dysfunction and normalization of NO generation

**Fig. 4:**
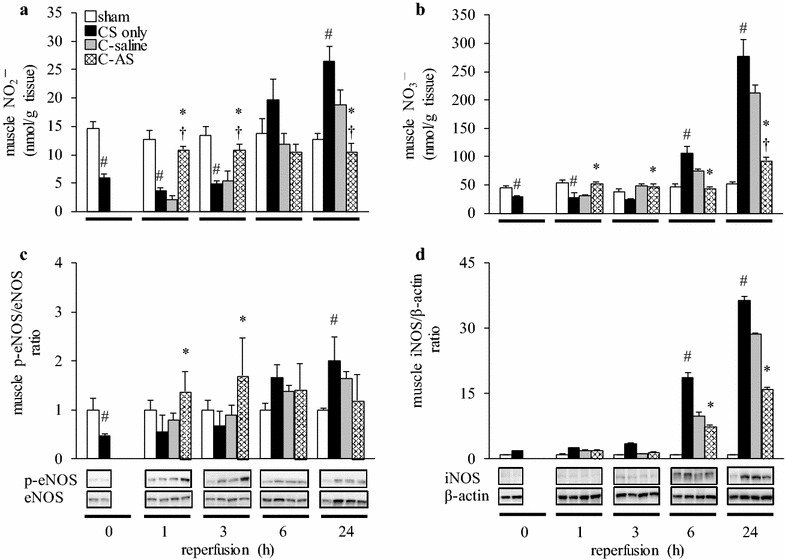
Effect of fluid resuscitation on NO mechanism in CS rats. **a** Muscle NO_2_^−^, **b** muscle NO_3_^−^, **c** muscle p-eNOS/eNOS ratio, and **d** muscle iNOS/β-actin ratio. *White bar* sham, *black bar* CS-only, *gray bar* C-saline, and *shaded bar* C-AS. Values represent mean ± SEM (*n* = 3). ^#^ *p* < 0.05 versus sham group, *p < 0.05 versus CS-only group, and ^†^ *p* < 0.05 versus C-saline group (Tukey’s test). *CS* crush syndrome, *NO* nitric oxide, *eNOS* endothelial nitric oxide synthase, *iNOS* inducible nitric oxide synthase

NO_2_^−^ and NO_3_^−^ concentrations in injured muscle were lower in the CS-only group after 0, 1, and 3 h, and higher after 24 h of reperfusion than those in the sham group (Fig. 4a, b). This resulted in a temporary decrease in eNOS phosphorylation (i.e., activity) and indicated that the subsequent increase in eNOS activity and iNOS expression were related (Fig. 4c, d). By contrast, NO_2_^−^ concentrations in the injured muscles of the C-AS group were similar to those in the sham group, and those in the C-saline group were not significantly different from those in the CS-only group. eNOS activity in the C-AS group was significantly increased compared to that in the other groups after 1 and 3 h of reperfusion. In addition, iNOS expression was significantly decreased after 24 h of reperfusion. These changes were not observed in the C-saline group.

**Fig. 5:**
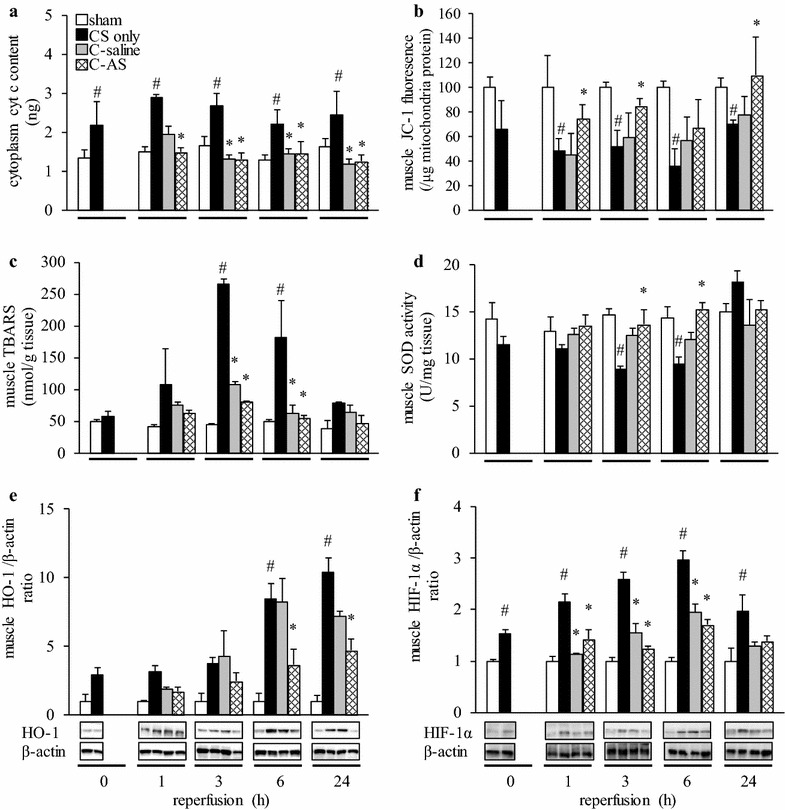
Effect of fluid resuscitation on mitochondrial function, antioxidant action, and low-oxygen injury in CS rats. **a** Cytoplasm cyt *c* content, **b** muscle JC-1 fluorescence, **c** muscle TBARS ratio, **d** muscle SOD activity, **e** muscle HO-1/β-actin ratio, and **f** muscle HIF-1α/β-actin ratio. *White bar* sham, *black bar* CS-only, *gray bar* C-saline and, *shaded bar* C-AS. Values represent mean ± SEM (*n* = 3). ^#^ *p* < 0.05 versus sham group, *p < 0.05 versus CS-only group, and ^†^ *p* < 0.05 versus C-saline group (Tukey’s test). *CS* crush syndrome, *cyt c* cytochrome *c*, *TBARS* thiobarbituric acid reactive substances, *SOD* superoxide dismutase, *HO-1* heme oxygenase-1, *HIF-1α* hypoxia-inducible factor-1 alpha

The most important factor in IRI is oxidative stress due to mitochondrial dysfunction. We measured cyt *c* leakage into the cytoplasm, JC-1 fluorescence intensity to determine MPT, TBARS to determine oxidative stress, and HIF-1α expression to evaluate tissue hypoxia (Fig. 5). We observed significantly increased cyt *c* leakage into the cytoplasm and significantly decreased JC-1 fluorescence intensity in the CS-only group compared to those in the sham group at 1, 3, 6, and 24 h (Fig. 5a, b). Simultaneously, the TBARS level in the CS-only group was significantly increased after 3 and 6 h of reperfusion (Fig. 5c). In contrast, SOD activity in the CS-only group was significantly decreased compared to that in the sham group after 3 and 6 h of reperfusion (Fig. 5d). Muscle HO-1 and HIF-1α expression in the CS-only group were significantly increased compared to those in the sham group (Fig. 5e, f). Accordingly, the RBC levels in the CS-only group tended to be higher than those in the sham group during the experimental period (Additional file 12: Table S9). The 50% inhibitory concentration (i.e., the direct radical scavenging ability) of AS was approximately 1/25,000 the value of AA (531.7 ± 190.6 vs. 0.02 ± 0.01 μM, respectively, Additional file 13: Fig. S4). No pathological changes were observed in the sham group. There was moderate neutrophil infiltration, moderate edema, mild degeneration, and atrophy in the CS-only group. Muscle edema and neutrophil infiltration were reduced in the C-AS group compared to that in the CS-only and sham groups after 6 and 24 h of reperfusion (Additional file 4: Table S2; Additional file 5: Fig. S3) (Fig. 5g, h). Overall, in the C-AS group, these pathologies improved compared to those in the CS-only and C-saline groups, and were similar to those in the sham group.

### Blood and urine concentration profile and PK parameters of AS

The urinary profile and PK parameters of the C-AS group were not significantly different from those of the sham-AS group, as determined by HPLC methods. A small quantity of urinary AS excretion was detected, but did not affect PK (data not shown). Therefore, LC–MS/MS was performed only for the plasma profile.

We determined the plasma concentration profile and PK parameters of AS in sham and CS rats, using LC–MS/MS methods. CAG was not detected in blood samples. The plasma concentration of the AS profile was similar between the sham-AS and C-AS groups 0.083–3 h after reperfusion. The profile of the C-AS group was significantly lower than that of the sham-AS group 4–24 h after administration (Additional file 14: Fig. S5). The PK parameters (AUC, MRT, *V*_ss_ *, T*_1/2_, and *T*_max_) of the C-AS group were significantly increased compared to those of the sham-AS group, and the CL_tot_ levels of the C-AS group were significantly decreased compared to those of the sham-AS group (Table 1).

**Table 1:** Plasma pharmacokinetic parameters of AS in sham and CS rats

	AUC_0–∞h_ (μg h/mL)	MRT (h)	CL_tot_ (mL/h)	*V*_ss_ (mL)	*K*_el_ (h^−1^)	*T*_1/2_ (h)	*T*_max_ (h)	*C*_max_ (μg/mL)
Sham-AS	32.9 ± 1.5	2.1 ± 0.0	84.4 ± 4.2	219 ± 41	0.80 ± 0.03	0.88 ± 0.04	1.83 ± 0.33	10.1 ± 0.4
C-AS	36.0 ± 1.3*	2.6 ± 0.2*	78.6 ± 3.4	244 ± 28	0.49 ± 0.02*	1.37 ± 0.03*	2.70 ± 0.11 *	10.3 ± 0.3

Values are presented as the mean ± SEM (*n* = 3–6)

*AUC* area under the concentration–time curve, *MRT* mean residence time, *CL*_*tot*_ total body clearance, *V*_*ss*_ steady-state volume of distribution, *K*_*el*_ elimination rate constant, *T*_*1/2*_ biological half-life, *T*_*max*_ time to maximum drug concentration, *C*_*max*_ maximum drug concentration, *AS* astragaloside-IV, *CS* crush syndrome

*p < 0.05 versus sham-AS group analyzed by Student’s t test

## Discussion

We performed this study to establish an AS bolus injection that could be used as a simple therapeutic method to treat CS to minimize severe symptoms and improve morbidity and mortality [17–20]. An AS bolus injection of 10 mg/kg improved the survival rate, but a 20 mg/kg AS bolus injection did not because metabolic and respiratory alkalosis developed in the 20 mg kg AS group more severely than in the 10 mg/kg AS group (Additional file 15: Fig. S6). Therefore, the beneficial AS effect is likely “bell-shaped.” However, using fluid resuscitation in addition to the optimal, 10 mg/kg AS dose is suggested to boost efficacy when treating CS.

Kidney injury can cause renal failure, which is a serious complication of CS that can result from circulatory shock, renal afferent arteriolar vasoconstriction (urinary concentration), increased urinary myoglobin levels, or metabolic acidosis (urinary acidity) [21–23]. All of these can cause precipitation in distal convoluted tubules and tubular cast formation with subsequent tubular obstruction. Myoglobin accumulation has also been shown to cause oxidative injury [24, 25]. In these cases, renal replacement therapy can improve patient survival. Normal and bicarbonate-containing saline are recommended as fluids for preventing hyperkalemia and circulatory shock associated with CS. Moreover, maintaining urinary pH above 6.5 can prevent intratubular deposition of myoglobin [26]. In the present study, administration of fluid containing AS significantly improved outcomes compared to administration of normal saline. In addition, the C-AS group showed an improvement in GFR, urinary pH, and volume compared to the CS-only group, indicating that treatment with AS protected against renal failure in CS. Interestingly, the NAG level in renal tubules, a marker of cytotoxicity, was normalized in the C-AS group compared to that in the CS-only group. Tan et al. [8] demonstrated that AS attenuated cystatin C, a kidney dysfunction marker, by suppressing nuclear factor kappa B (NF-κB) expression in the kidney of rats with IRI-induced acute kidney injury. Generally, HO-1 is induced by NF-κB, and is cytoprotective following an IRI. However, HO-1 protein expression in the C-AS group was lower than that in the CS-only and C-saline groups in the kidney. The reason for this is likely that HO-1 protein was not needed following IRI or that HO-1 protein expression was inhibited. The improved kidney function observed in this study resulted from the former reason. We evaluated blood NAG concentration as a new kidney function marker. NAG is a lysosomal enzyme in the renal proximal tubular epithelium cells that can be released when the tubules in the kidney are damaged. Moreover, NAG is found in many tissues in the body and has been reported previously to be detectable in serum [27]. In the clinical situation, urine sample collection from CS patients is more difficult than blood sampling because CS patients develop anuria. The blood NAG concentration was much lower than its urine concentration. Nevertheless, we suggest that blood NAG can be used to assess effectiveness of reperfusion at 24 h (Fig. 2d).

CS is known as rescue death because up to 20% of deaths occur shortly after extrication [28]. In this study, blood K^+^ levels in the CS-only group were higher than those in the sham group, particularly over time, increasing the risk of cardiac arrest in the CS-only group. We previously demonstrated the development of cardiac arrest in CS model rats [12]. Interestingly, the decreasing K^+^ levels in the group administered AS-containing fluid were related to the improvement in AKI and low CPK levels during the early experimental period, which is possibly related to reduced muscle damage. The administration of AS-containing fluid likely normalized hyperkalemia via classical mechanisms such as renal excretion of K^+^ and cellular uptake of K^+^. Moreover, our data suggest that AS dramatically improved mitochondrial dysfunction following myocytolysis and had anti-inflammatory effects following IRI. First, the administration of AS-containing fluid prevented mitochondrial damage by improving mitochondrial membrane potential and mitochondrial outer membrane function (Fig. 5a, b), because a direct radical scavenging ability of AS was not observed (Additional file 13: Fig. S4). Generally, under ischemic (hypoxic) conditions, mitochondrial dysfunction results in ROS generation and NO protects mitochondria by acting as an oxygen radical scavenger [29]. However, in this study, the anti-radical effects of AS activated eNOS and improved the decreased SOD activity caused by NO. Interestingly, the TBARS level and SOD activity in the CS-only group were significantly decreased compared to those in the sham group 3 and 6 h after reperfusion. iNOS expression increased and the TBARS level decreased in injured muscles 24 h after reperfusion. Moreover, these results demonstrated that increasing HIF-1α expression and RBC levels during the experimental period generated little NO via the NOS systems because of insufficient oxygen in the injured muscle. Therefore, radical injury in CS was not worsened by iNOS overexpression, but by mitochondrial dysfunction and decreased SOD activity. Second, AS-containing fluid inhibited leukocyte infiltration, as indicated by MPO activity and pathological changes due to decreased blood IL-1β and TNF-α levels. NO is an important regulatory factor that inhibits leukocyte adhesion [30], expression of intercellular adhesion molecules, and p-selectin [31]. We previously reported that improving NO consumption attenuated the vascular endothelial damage [10, 11, 16]. Therefore, AS may have a similar mechanism as we previously reported because eNOS was activated in the C-AS group during the early phase. Interestingly, the cardio-circulatory dynamics of the C-AS group were improved more than those of the sham group because edema was improved via attenuated vascular endothelial damage. In this study, the WBC levels of the CS group were markedly decreased after a reaching a maximum at 3–24 h. The continued rise of blood MPO activity (indicative of leukocyte numbers) suggested an inflammatory response, which could subsequently result in an immunocompromised state (Fig. 3b; Additional file 12: Table S9). According to Gunner [32], sepsis and wound infection were found in approximately 50% of cases that developed into CS. Therefore, we suggest that improved immunosuppression was due to an excessive inflammatory response following massive resuscitation with AS-containing fluid.

CS rats displayed characteristic PK of impaired drug metabolism (i.e., inhibition of liver drug-metabolizing enzymes and the formation of the third space) [33], but we conclude that the high blood concentration of AS (Additional file 13: Fig. S4) occurred because AS was not renally excreted in CS rats [renal clearance (CL_r_)/CL_tot_ ratio: 0.159 ± 0.018/0.180 ± 0.012, as measured by HPLC].

## Conclusion

Administration of 10 mg/kg AS-containing fluid led to a dramatic improvement in survival following CS owing to direct and indirect anti-oxidative effects of AS in the kidney, and the prevention of mitochondrial dysfunction and inflammation owing to AS acting as an NO donor in injured muscle (Additional file 15: Fig. S6). Moreover, these findings are important with respect to the fluid resuscitation limitation of worsening edema by fluid resuscitation for prevention of kidney failure and cardiac arrest and the promising therapeutic strategy of prevention of sepsis by immunosuppression via excessive inflammatory response.

## Additional files

**Additional file 1: Fig. S1.** Chemical structure of astragaloside-IV.

**Additional file 2: Table S1.** Effect of fluid resuscitation on blood gas parameters in CS rats.

**Additional file 3: Fig. S2.** Effect of single injection on MBP level in CS rats. White bar, sham; black bar, CS-only; and shaded bar, 10 AS. The 10 AS group 24 h after reperfusion was not observed because all animals in the 10 AS group died. Values represent mean ± SEM (n = 3). ^#^ *p* < 0.05 versus sham group and *p < 0.05 versus CS-only group (Tukey’s test).

**Additional file 4: Table S2.** Effect of fluid resuscitation on HR, SBP, and DBP levels in CS rats.

**Additional file 5: Fig. S3.** Effect of fluid resuscitation on pathological changes in the muscle of CS rats. Micrographs are representative of three independent experiments (200× magnification; scale bars = 100 μm).

**Additional file 6: Table S3.** Effect of fluid resuscitation on edema in CS rats.

**Additional file 7: Table S4.** Effect of fluid resuscitation on blood gas parameters in CS rats.

**Additional file 8: Table S5.** Effect of fluid resuscitation on lactate in CS rats.

**Additional file 9: Table S6.** Effect of fluid resuscitation on kidney function parameters by blood sample in CS rats.

**Additional file 10: Table S7.** Effect of fluid resuscitation on kidney function parameters by urine sample in CS rats.

**Additional file 11: Table S8.** Effect of fluid resuscitation on WBC and platelets in CS rats.

**Additional file 12: Table S9.** Effect of fluid resuscitation on RBC in CS rats.

**Additional file 13: Fig. S4.** DPPH radical scavenging assay for AS. White bar, AA; black bar; AS. AA (0.01, 0.05, 0.1, 0.25, 0.5, 1.0, 2.5, 5, and 10 μM), AS-containing methanol solution (0.1, 0.5, 1.0, 10, 100, 1000, 2000, and 3000 μM) or methanol was added to 10 μM DPPH and the reaction mixture (AA, AS methanol (without AA or AS; M) solution/10 μM DPPH = 1/1; i.e., AA _517 nm_, AS _517 nm_, and M _517 nm_) was kept at 30 °C for 30 min. The absorbance was measured at a wavelength of 517 nm. The rate of DPPH radical scavenging activity was calculated using the following formula: [1- (AA or AS_517 nm_ – methanol blank _517 nm_)/(M _517 nm_ - methanol blank _517 nm_)] × 100. Values represent mean ± SEM (n = 3). *p < 0.05 versus AA group (Student’s *t* test).

**Additional file 14: Fig. S5.** Plasma AS concentration profile in sham and CS rats. (A) sham-AS, (B) C-AS, (C) semi-log scale. White circle, sham-AS and black circle, C-AS. Values represent mean ± SEM (n = 3-6). *p < 0.05 versus sham-AS group (Student’s t-test).

**Additional file 15: Fig. S6.** Schematic summary diagram of physiological changes caused by CS.

## Abbreviations

CS: crush syndrome
AKI: acute kidney injury
ARDS: acute respiratory distress syndrome
SIRS: systemic inflammatory response syndrome
IRI: ischemia/reperfusion injury
AS: astragaloside-IV
NO: nitric oxide
eNOS: endothelial nitric oxide synthase
DPPH: 1,1-diphenyl-2-picrylhydrazyl
AA: ascorbic acid
MBP: mean blood pressure
*Pa*O_2_: partial pressure of oxygen
*Pa*CO_2_: partial pressure of carbon dioxide
HCO_3_^−^: bicarbonate
BUN: blood urea nitrogen
Cre: creatinine
CPK: creatine phosphokinase
RBC: red blood cells
WBC: white blood cells
IL-1β: interleukin-1 beta
TNF-α: tumor necrosis factor alpha
GFR: glomerular filtration rate
NAG: *N*-acetyl-β-d-glucosaminidase
KIM-1: kidney injury marker-1
ROS: reactive oxygen species
TBARS: thiobarbituric acid reactive substances
MPO: myeloperoxidase
MPT: mitochondrial permeability transition
cyt *c*: cytochrome *c*
SOD: superoxide dismutase
iNOS: inducible nitric oxide synthase
HO-1: heme oxygenase-1
HIF-1α: hypoxia-inducible factor-1 alpha
PK: pharmacokinetic
DIG: digoxin
AUC: area under the curve
MRT: mean residence time
CL_tot_: total body clearance
CL_r_: renal clearance
*V*_ss_: steady-state volume of distribution
*K*_el_: elimination rate constant
*T*_1/2_: biological half-life
*T*_max_: maximum drug concentration time
*C*_max_: maximum blood concentration
ESI: electrospray ionization
NO_2_^−^: nitrite
NO_3_^−^: nitrate
NF-ĸB: nuclear factor kappa B
