# Supplementary material for: Astragaloside-IV prevents acute kidney injury and inflammation by normalizing muscular mitochondrial function associated with a nitric oxide protective mechanism in crush syndrome rats
Source: Ann Intensive Care. 2017 Sep 4;7:90. doi: 10.1186/s13613-017-0313-2 (PMC5583140; doi:10.1186/s13613-017-0313-2)
Supplement: Supplementary file 2 — Additional file 2: Table S1. Effect of fluid resuscitation on blood gas parameters in CS rats. [file 13613_2017_313_MOESM2_ESM.docx]

| **SUPPLEMENTAL DIGITAL CONTENT Table 1. Effect of fluid resuscitation on blood gas parameters in CS rats.** | | | | | | | | | | | | | | | | | | | | | | | | | | | | | | | | | | | | | |  |
| --- | --- | --- | --- | --- | --- | --- | --- | --- | --- | --- | --- | --- | --- | --- | --- | --- | --- | --- | --- | --- | --- | --- | --- | --- | --- | --- | --- | --- | --- | --- | --- | --- | --- | --- | --- | --- | --- | --- |
|  |  | reperfusion (h) | | | | | | | | | | | | | | | | | | | | | | | | | | | | | | | | | | | |  |
|  |  | 0 | | |  | 1 | | | | | |  | | 3 | | | | | |  | | 6 | | | | | |  | | 24 | | | | | |  | |  |
| pH | sham | 7.42 | ± | 0.01 |  | 7.43 | | ± | | 0.01 | |  | | 7.45 | | ± | | 0.03 | |  | | 7.46 | | ± | | 0.02 | |  | | 7.44 | | ± | | 0.02 | |  | |  |
|  | CS only | 7.40 | ± | 0.03 |  | 7.35 | | ± | | 0.03 | | ^#^ | | 7.30 | | ± | | 0.03 | | ^#^ | | 7.28 | | ± | | 0.01 | | ^#^ | | 7.26 | | ± | | 0.03 | | ^#^ | |  |
|  | C-10 AS-one | 7.41 |  | 0.01 |  | 7.40 | |  | | 0.01 | |  | | 7.43 | |  | | 0.01 | |  | | 7.40 | |  | | 0.03 | |  | |  | |  | |  | |  | |  |
|  | C-20 AS-one | 7.42 |  | 0.01 |  | 7.45 | |  | | 0.00 | |  | | 7.50 | |  | | 0.00 | |  | | 7.48 | |  | | 0.01 | |  | |  | |  | |  | |  | |  |
| *P*aCO_2_ | sham | 39.4 | ± | 1.0 |  | 41.3 | | ± | | 0.4 | |  | | 40.0 | | ± | | 0.3 | |  | | 41.0 | | ± | | 1.5 | |  | | 43.2 | | ± | | 1.3 | |  | |  |
|  | CS only | 36.2 | ± | 1.6 |  | 34.2 | | ± | | 0.6 | |  | | 37.2 | | ± | | 0.9 | |  | | 32.0 | | ± | | 0.7 | | ^#^ | | 31.4 | | ± | | 0.4 | | ^#^ | |  |
| (mmHg) | C-10 AS-one | 38.6 |  | 0.8 |  | 34.3 | |  | | 0.9 | |  | | 27.3 | |  | | 1.5 | |  | | 23.0 | |  | | 3.1 | |  | |  | |  | |  | |  | |  |
|  | C-20 AS-one | 37.0 |  | 0.3 |  | 31.0 | |  | | 0.6 | |  | | 22.6 | |  | | 1.3 | |  | | 17.7 | |  | | 1.6 | |  | |  | |  | |  | |  | |  |
| *P*aO_2_ | sham | 85.4 | ± | 2.8 |  | 82.9 | | ± | | 1.9 | |  | | 80.3 | | ± | | 4.1 | |  | | 84.4 | | ± | | 3.4 | |  | | 86.3 | | ± | | 1.3 | |  | |  |
|  | CS only | 80.9 | ± | 4.3 |  | 90.9 | | ± | | 2.8 | |  | | 99.3 | | ± | | 5.1 | | ^#^ | | 97.4 | | ± | | 5.1 | | ^#^ | | 111.4 | | ± | | 9.6 | | ^#^ | |  |
| (mmHg) | C-10 AS-one | 83.6 |  | 3.2 |  | 92.7 | |  | | 1.2 | |  | | 114.3 | |  | | 4.1 | |  | | 121.7 | |  | | 3.2 | |  | |  | |  | |  | |  | |  |
|  | C-20 AS-one | 80.6 |  | 3.7 |  | 102.3 | |  | | 3.2 | |  | | 122.7 | |  | | 2.3 | |  | | 132.3 | |  | | 1.5 | |  | |  | |  | |  | |  | |  |
| BE | sham | 1.9 | ± | 0.1 |  | 2.5 | | ± | | 0.2 | |  | | 2.8 | | ± | | 0.4 | |  | | 2.5 | | ± | | 0.9 | |  | | 2.9 | | ± | | 0.9 | |  | |  |
|  | CS only | 2.0 | ± | 0.1 |  | -3.5 | | ± | | 0.6 | | ^#^ | | -5.5 | | ± | | 0.8 | | ^#^ | | -10.3 | | ± | | 0.2 | | ^#^ | | -9.8 | | ± | | 0.9 | | ^#^ | |  |
| (mmol/L) | C-10 AS-one | 1.8 |  | 0.5 |  | -2.3 | |  | | 0.9 | |  | | -6.0 | |  | | 0.6 | |  | | -10.7 | |  | | 0.3 | |  | |  | |  | |  | |  | |  |
|  | C-20 AS-one | 2.0 |  | 0.6 |  | -3.7 | |  | | 0.7 | |  | | -7.0 | |  | | 1.2 | |  | | -14.0 | |  | | 1.0 | |  | |  | |  | |  | |  | |  |
| HCO_3_^－^ | sham | 26.4 | ± | 0.1 |  | 28.7 | | ± | | 0.2 | |  | | 25.3 | | ± | | 0.2 | |  | | 28.5 | | ± | | 0.3 | |  | | 27.3 | | ± | | 0.4 | |  | |  |
|  | CS only | 24.4 | ± | 0.8 |  | 22.6 | | ± | | 0.5 | | ^#^ | | 20.3 | | ± | | 0.2 | | ^#^ | | 14.2 | | ± | | 0.8 | | ^#^ | | 16.8 | | ± | | 0.5 | | ^#^ | |  |
| (mEq/L) | C-10 AS-one | 26.3 |  | 0.7 |  | 24.3 | |  | | 0.9 | |  | | 18.2 | |  | | 0.7 | |  | | 14.2 | |  | | 0.9 | |  | |  | |  | |  | |  | |  |
|  | C-20 AS-one | 26.7 |  | 1.0 |  | 22.3 | |  | | 0.9 | |  | | 16.8 | |  | | 0.5 | |  | | 13.2 | |  | | 0.6 | |  | |  | |  | |  | |  | |  |
| Lactate  (mmol/L) | sham | 1.0 | ± | 0.1 |  | | 0.8 | | ± | | 0.1 | |  | | 0.9 | | ± | | 0.1 | |  | | 0.7 | | ± | | 0.3 | |  | | 0.5 | | ± | | 0.0 | |  | |
|  | CS only | 0.8 | ± | 0.2 |  | | 1.1 | | ± | | 0.3 | |  | | 1.9 | | ± | | 0.1 | | # | | 2.6 | | ± | | 0.1 | | # | | 3.3 | | ± | | 0.3 | | # | |
|  | C-10 AS-one | 0.5 |  | 0.3 |  | | 1.1 | |  | | 0.1 | |  | | 1.9 | |  | | 0.5 | |  | | 2.3 | |  | | 0.2 | |  | |  | |  | |  | |  | |
|  | C-20 AS-one | 0.8 |  | 0.5 |  | | 1.2 | |  | | 0.1 | |  | | 1.9 | |  | | 0.1 | |  | | 2.6 | |  | | 0.2 | |  | |  | |  | |  | |  | |
| Values represent mean ± SEM (n = 3-6 each). ^#^P < 0.05 vs. sham group; ^*^P < 0.05 vs. CS-only group; ^†^P < 0.05 vs. C-saline group (Tukey's test).　Measurement of C-10 and 20 AS-one groups were not performed because rats dead at before reperfusion 24 h before. | | | | | | | | | | | | | | | | | | | | | | | | | | | | | | | | | | | | | |  |
|  |  |  |  |  |  |  |  |  |  |  |  |  |  |  |  |  |  |  |  |  |  |  |  |  |  |  |  |  |  |  |  |  |  |  |  |  |  |  |
