# Supplementary material for: Astragaloside-IV prevents acute kidney injury and inflammation by normalizing muscular mitochondrial function associated with a nitric oxide protective mechanism in crush syndrome rats
Source: Ann Intensive Care. 2017 Sep 4;7:90. doi: 10.1186/s13613-017-0313-2 (PMC5583140; doi:10.1186/s13613-017-0313-2)
Supplement: Supplementary file 4 — Additional file 4: Table S2. Effect of fluid resuscitation on HR, SBP, and DBP levels in CS rats. [file 13613_2017_313_MOESM4_ESM.docx]

| **SUPPLEMENTAL DIGITAL CONTENT Table 2. Effect of fluid resuscitation on HR, SBP, and DBP levels in CS rats.** | | | | | | | | | | | | | | | | | | | | | |
| --- | --- | --- | --- | --- | --- | --- | --- | --- | --- | --- | --- | --- | --- | --- | --- | --- | --- | --- | --- | --- | --- |
|  |  |  |  |  |  |  |  |  |  |  |  |  |  |  |  |  |  |  |  |  |  |
|  |  | reperfusion (h) | | | | | | | | | | | | | | | | | | | |
|  |  | 0 | | |  | 1 | | |  | 3 | | |  | 6 | | |  | 24 | | |  |
| HR | sham | 389 | ± | 10 |  | 416 | ± | 21 |  | 400 | ± | 15 |  | 414 | ± | 17 |  | 424 | ± | 51 |  |
|  | CS only | 399 | ± | 19 |  | 356 | ± | 13 |  | 340 | ± | 24 | ^#^ | 301 | ± | 18 | ^#^ | 250 | ± | 49 | ^#^ |
| (bpm) | C-saline | 402 | ± | 16 |  | 398 | ± | 36 |  | 387 | ± | 14 |  | 352 | ± | 10 |  | 363 | ± | 3 | ^*^ |
|  | C-AS | 415 | ± | 9 |  | 300 | ± | 24 | ^*^ | 186 | ± | 20 | ^*,†^ | 276 | ± | 56 | ^†^ | 302 | ± | 62 | ^†^ |
| SBP | sham | 136 | ± | 2 |  | 139 | ± | 2 |  | 124 | ± | 2 |  | 130 | ± | 5 |  | 137 | ± | 4 |  |
|  | CS only | 128 | ± | 6 |  | 109 | ± | 3 | ^#^ | 83 | ± | 10 | ^#^ | 94 | ± | 5 | ^#^ | 89 | ± | 2 | ^#^ |
| (mmHg) | C-saline | 120 | ± | 9 |  | 123 | ± | 6 |  | 110 | ± | 2 |  | 108 | ± | 5 | ^*^ | 107 | ± | 4 | ^*^ |
|  | C-AS | 129 | ± | 7 |  | 136 | ± | 5 |  | 115 | ± | 3 |  | 107 | ± | 4 | ^*^ | 109 | ± | 3 | ^*^ |
| DBP | sham | 110 | ± | 3 |  | 113 | ± | 1 |  | 100 | ± | 4 |  | 102 | ± | 4 |  | 114 | ± | 5 |  |
|  | CS only | 99 | ± | 3 |  | 79 | ± | 4 | ^#^ | 65 | ± | 11 | ^#^ | 65 | ± | 5 | ^#^ | 61 | ± | 2 | ^#^ |
| (mmHg) | C-saline | 96 | ± | 2 |  | 101 | ± | 3 | ^*^ | 91 | ± | 4 | ^*^ | 92 | ± | 4 | ^*^ | 97 | ± | 4 | ^*^ |
|  | C-AS | 96 | ± | 3 |  | 105 | ± | 1 | ^*^ | 107 | ± | 3 | ^*^ | 95 | ± | 2 | ^*^ | 90 | ± | 7 | ^*^ |
| Values represent mean ± SEM (n = 3-6 each). ^#^P < 0.05 vs. sham group; *P < 0.05 vs. CS-only group; ^†^P < 0.05 vs. C-saline group (Tukey's test). | | | | | | | | | | | | | | | | | | | | | |
|  |  |  |  |  |  |  |  |  |  |  |  |  |  |  |  |  |  |  |  |  |  |
