# Supplementary material for: Astragaloside-IV prevents acute kidney injury and inflammation by normalizing muscular mitochondrial function associated with a nitric oxide protective mechanism in crush syndrome rats
Source: Ann Intensive Care. 2017 Sep 4;7:90. doi: 10.1186/s13613-017-0313-2 (PMC5583140; doi:10.1186/s13613-017-0313-2)
Supplement: Supplementary file 6 — Additional file 6: Table S3. Effect of fluid resuscitation on edema in CS rats. [file 13613_2017_313_MOESM6_ESM.docx]

| **SUPPLEMENTAL DIGITAL CONTENT Table 3. Effect of fluid resuscitation on edema in CS rats.** | | | | | | | | | | | | | | | | | | | | | |
| --- | --- | --- | --- | --- | --- | --- | --- | --- | --- | --- | --- | --- | --- | --- | --- | --- | --- | --- | --- | --- | --- |
|  |  |  |  |  |  |  |  |  |  |  |  |  |  |  |  |  |  |  |  |  |  |
|  |  | reperfusion (h) | | | | | | | | | | | | | | | | | | | |
|  |  | 0 | | |  | 1 | | |  | 3 | | |  | 6 | | |  | 24 | | |  |
| gastrocnemius muscle | sham | 1.02 | ± | 0.02 |  | 1.15 | ± | 0.01 |  | 1.20 | ± | 0.02 |  | 1.10 | ± | 0.01 |  | 1.05 | ± | 0.05 |  |
|  | CS only | 1.18 | ± | 0.01 | ^#^ | 1.30 | ± | 0.05 | ^#^ | 1.38 | ± | 0.02 | ^#^ | 1.59 | ± | 0.03 | ^#^ | 1.78 | ± | 0.02 | ^#^ |
| (g/100 g B.W.) | C-saline | 1.09 | ± | 0.03 |  | 1.50 | ± | 0.02 | ^*^ | 1.78 | ± | 0.04 | ^*^ | 1.60 | ± | 0.03 | ^*^ | 1.69 | ± | 0.04 |  |
|  | C-AS | 1.12 | ± | 0.05 |  | 1.56 | ± | 0.01 | ^*^ | 1.60 | ± | 0.03 | ^*^ | 1.63 | ± | 0.02 | ^*^ | 1.68 | ± | 0.03 |  |
| Values represent mean ± SEM (n = 3-6 each). ^#^P < 0.05 vs. sham group; *P < 0.05 vs. CS-only group; ^†^P < 0.05 vs. C-saline group (Tukey's test). | | | | | | | | | | | | | | | | | | | | | |
|  |  |  |  |  |  |  |  |  |  |  |  |  |  |  |  |  |  |  |  |  |  |
