# Supplementary material for: Astragaloside-IV prevents acute kidney injury and inflammation by normalizing muscular mitochondrial function associated with a nitric oxide protective mechanism in crush syndrome rats
Source: Ann Intensive Care. 2017 Sep 4;7:90. doi: 10.1186/s13613-017-0313-2 (PMC5583140; doi:10.1186/s13613-017-0313-2)
Supplement: Supplementary file 7 — Additional file 7: Table S4. Effect of fluid resuscitation on blood gas parameters in CS rats. [file 13613_2017_313_MOESM7_ESM.docx]

| **SUPPLEMENTAL DIGITAL CONTENT Table 4. Effect of fluid resuscitation on blood gas parameters in CS rats.** | | | | | | | | | | | | | | | | | | | | | |
| --- | --- | --- | --- | --- | --- | --- | --- | --- | --- | --- | --- | --- | --- | --- | --- | --- | --- | --- | --- | --- | --- |
|  |  | reperfusion (h) | | | | | | | | | | | | | | | | | | | |
|  |  | 0 | | |  | 1 | | |  | 3 | | |  | 6 | | |  | 24 | | |  |
| pH | sham | 7.42 | ± | 0.01 |  | 7.43 | ± | 0.01 |  | 7.45 | ± | 0.03 |  | 7.46 | ± | 0.02 |  | 7.44 | ± | 0.02 |  |
|  | CS only | 7.40 | ± | 0.03 |  | 7.35 | ± | 0.03 | ^#^ | 7.30 | ± | 0.03 | ^#^ | 7.28 | ± | 0.01 | ^#^ | 7.26 | ± | 0.03 | ^#^ |
|  | C-saline | 7.41 | ± | 0.01 |  | 7.35 | ± | 0.03 |  | 7.31 | ± | 0.04 |  | 7.32 | ± | 0.01 |  | 7.35 | ± | 0.05 |  |
|  | C-AS | 7.44 | ± | 0.02 |  | 7.34 | ± | 0.03 |  | 7.37 | ± | 0.01 |  | 7.31 | ± | 0.01 |  | 7.45 | ± | 0.01 | ^*,†^ |
| *P*aCO_2_ | sham | 39.4 | ± | 1.0 |  | 41.3 | ± | 0.4 |  | 40.0 | ± | 0.3 |  | 41.0 | ± | 1.5 |  | 43.2 | ± | 1.3 |  |
|  | CS only | 36.2 | ± | 1.6 |  | 34.2 | ± | 0.6 |  | 37.2 | ± | 0.9 |  | 32.0 | ± | 0.7 | ^#^ | 31.4 | ± | 0.4 | ^#^ |
| (mmHg) | C-saline | 37.1 | ± | 1.5 |  | 33.8 | ± | 0.8 |  | 35.3 | ± | 0.9 |  | 36.3 | ± | 0.8 |  | 35.3 | ± | 1.3 |  |
|  | C-AS | 35.3 | ± | 1.4 |  | 31.6 | ± | 1.2 |  | 30.3 | ± | 1.2 | ^*^ | 36.0 | ± | 1.5 |  | 29.2 | ± | 0.9 |  |
| *P*aO_2_ | sham | 85.4 | ± | 2.8 |  | 82.9 | ± | 1.9 |  | 80.3 | ± | 4.1 |  | 84.4 | ± | 3.4 |  | 86.3 | ± | 1.3 |  |
|  | CS only | 80.9 | ± | 4.3 |  | 90.9 | ± | 2.8 |  | 99.3 | ± | 5.1 | ^#^ | 97.4 | ± | 5.1 | ^#^ | 111.4 | ± | 9.6 | ^#^ |
| (mmHg) | C-saline | 88.1 | ± | 3.6 |  | 95.5 | ± | 5.3 |  | 98.1 | ± | 5.1 |  | 95.3 | ± | 4.8 |  | 110.8 | ± | 3.7 |  |
|  | C-AS | 86.3 | ± | 4.4 |  | 100.3 | ± | 8.3 |  | 104.5 | ± | 10.4 |  | 112.2 | ± | 4.4 |  | 125.5 | ± | 6.7 |  |
| BE | sham | 1.9 | ± | 0.1 |  | 2.5 | ± | 0.2 |  | 2.8 | ± | 0.4 |  | 2.5 | ± | 0.9 |  | 2.9 | ± | 0.9 |  |
|  | CS only | 2.0 | ± | 0.1 |  | -3.5 | ± | 0.6 | ^#^ | -5.5 | ± | 0.8 | ^#^ | -10.3 | ± | 0.2 | ^#^ | -9.8 | ± | 0.9 | ^#^ |
| (mmol/L) | C-saline | 3.5 | ± | 0.2 |  | -2.0 | ± | 0.8 |  | -6.0 | ± | 0.3 |  | -8.5 | ± | 1.2 |  | -6.8 | ± | 0.6 | ^*^ |
|  | C-AS | 2.8 | ± | 0.3 |  | -3.3 | ± | 0.5 |  | -7.8 | ± | 1.3 |  | -9.2 | ± | 1.1 |  | -1.3 | ± | 0.3 | ^*,†^ |
| HCO_3_^－^ | sham | 26.4 | ± | 0.1 |  | 28.7 | ± | 0.2 |  | 25.3 | ± | 0.2 |  | 28.5 | ± | 0.3 |  | 27.3 | ± | 0.4 |  |
|  | CS only | 24.4 | ± | 0.8 |  | 22.6 | ± | 0.5 | ^#^ | 20.3 | ± | 0.2 | ^#^ | 14.2 | ± | 0.8 | ^#^ | 16.8 | ± | 0.5 | ^#^ |
| (mEq/L) | C-saline | 25.6 | ± | 0.2 |  | 20.9 | ± | 0.6 |  | 19.3 | ± | 0.2 |  | 15.6 | ± | 0.2 |  | 18.8 | ± | 1.3 |  |
|  | C-AS | 24.9 | ± | 0.3 |  | 21.6 | ± | 0.4 |  | 17.7 | ± | 1.0 |  | 17.4 | ± | 0.8 |  | 21.9 | ± | 1.4 | ^*^ |
| Values represent mean ± SEM (n = 3-6 each). ^#^P < 0.05 vs. sham group; ^*^P < 0.05 vs. CS-only group; ^†^P < 0.05 vs. C-saline group (Tukey's test). | | | | | | | | | | | | | | | | | | | | | |
|  |  |  |  |  |  |  |  |  |  |  |  |  |  |  |  |  |  |  |  |  |  |
