# Supplementary material for: Astragaloside-IV prevents acute kidney injury and inflammation by normalizing muscular mitochondrial function associated with a nitric oxide protective mechanism in crush syndrome rats
Source: Ann Intensive Care. 2017 Sep 4;7:90. doi: 10.1186/s13613-017-0313-2 (PMC5583140; doi:10.1186/s13613-017-0313-2)
Supplement: Supplementary file 8 — Additional file 8: Table S5. Effect of fluid resuscitation on lactate in CS rats. [file 13613_2017_313_MOESM8_ESM.docx]

| **SUPPLEMENTAL DIGITAL CONTENT Table 5. Effect of fluid resuscitation on lactate in CS rats.** | | | | | | | | | | | | | | | | | | | | | |
| --- | --- | --- | --- | --- | --- | --- | --- | --- | --- | --- | --- | --- | --- | --- | --- | --- | --- | --- | --- | --- | --- |
|  |  | reperfusion (h) | | | | | | | | | | | | | | | | | | | |
|  |  | 0 | | |  | 1 | | |  | 3 | | |  | 6 | | |  | 24 | | |  |
| lactate | sham | 1.0 | ± | 0.1 |  | 0.8 | ± | 0.1 |  | 0.9 | ± | 0.1 |  | 0.7 | ± | 0.3 |  | 0.5 | ± | 0.0 |  |
|  | CS only | 0.8 | ± | 0.2 |  | 1.1 | ± | 0.3 |  | 1.9 | ± | 0.1 | ^#^ | 2.6 | ± | 0.1 | ^#^ | 3.3 | ± | 0.3 | ^#^ |
| (mmol/L) | C-saline | 0.5 | ± | 0.2 |  | 0.8 | ± | 0.2 |  | 1.6 | ± | 0.1 |  | 2.1 | ± | 0.3 |  | 2.0 | ± | 0.7 |  |
|  | C-AS | 0.6 | ± | 0.1 |  | 1.0 | ± | 0.2 |  | 1.6 | ± | 0.4 |  | 1.8 | ± | 0.5 |  | 1.7 | ± | 0.2 |  |
| Values represent mean ± SEM (n = 3-6 each). ^#^P < 0.05 vs. sham group (Tukey's test). | | | | | | | | | | | | | | | | | | | | | |
|  |  |  |  |  |  |  |  |  |  |  |  |  |  |  |  |  |  |  |  |  |  |
