# Supplementary material for: Astragaloside-IV prevents acute kidney injury and inflammation by normalizing muscular mitochondrial function associated with a nitric oxide protective mechanism in crush syndrome rats
Source: Ann Intensive Care. 2017 Sep 4;7:90. doi: 10.1186/s13613-017-0313-2 (PMC5583140; doi:10.1186/s13613-017-0313-2)
Supplement: Supplementary file 9 — Additional file 9: Table S6. Effect of fluid resuscitation on kidney function parameters by blood sample in CS rats. [file 13613_2017_313_MOESM9_ESM.docx]

| **SUPPLEMENTAL DIGITAL CONTENT Table 6. Effect of fluid resuscitation on kidney function parameters by blood sample in CS rats.** | | | | | | | | | | | | | | | | | | | | | |
| --- | --- | --- | --- | --- | --- | --- | --- | --- | --- | --- | --- | --- | --- | --- | --- | --- | --- | --- | --- | --- | --- |
|  |  |  |  |  |  |  |  |  |  |  |  |  |  |  |  |  |  |  |  |  |  |
|  |  | reperfusion (h) | | | | | | | | | | | | | | | | | | | |
|  |  | 0 | | |  | 1 | | |  | 3 | | |  | 6 | | |  | 24 | | |  |
| BUN | sham | 20.6 | ± | 1.7 |  | 21.3 | ± | 1.6 |  | 13.5 | ± | 1.6 |  | 11.7 | ± | 1.1 |  | 20.4 | ± | 1.4 |  |
|  | CS only | 21.0 | ± | 3.9 |  | 28.3 | ± | 5.6 |  | 41.3 | ± | 4.5 | ^#^ | 56.6 | ± | 2.5 | ^#^ | 37.0 | ± | 1.9 | ^#^ |
| (mg/dL) | C-saline | 25.1 | ± | 3.6 |  | 15.9 | ± | 2.3 | ^*^ | 26.7 | ± | 0.6 |  | 34.2 | ± | 10.0 |  | 17.3 | ± | 1.2 | ^*^ |
|  | C-AS | 18.2 | ± | 4.0 |  | 17.2 | ± | 1.9 | ^*^ | 18.0 | ± | 0.3 | ^*^ | 23.8 | ± | 1.3 | ^*^ | 16.5 | ± | 0.4 | ^*^ |
| Cre | sham | 0.3 | ± | 0.1 |  | 0.3 | ± | 0.3 |  | 0.2 | ± | 0.0 |  | 0.2 | ± | 0.0 |  | 0.2 | ± | 0.0 |  |
|  | CS only | 0.4 | ± | 0.3 |  | 1.0 | ± | 0.2 |  | 1.5 | ± | 0.4 | ^#^ | 0.8 | ± | 0.1 | ^#^ | 0.5 | ± | 0.1 |  |
| (mg/dL) | C-saline | 0.5 | ± | 0.1 |  | 0.3 | ± | 0.0 |  | 0.2 | ± | 0.0 | ^*^ | 0.3 | ± | 0.1 | ^*^ | 0.4 | ± | 0.1 |  |
|  | C-AS | 0.4 | ± | 0.1 |  | 0.3 | ± | 0.1 |  | 0.3 | ± | 0.0 | ^*^ | 0.3 | ± | 0.0 | ^*^ | 0.3 | ± | 0.0 |  |
| Values represent mean ± SEM (n = 3-6 each). ^#^P < 0.05 vs. sham group; ^*^P < 0.05 vs. CS-only group; ^†^P < 0.05 vs. C-saline group (Tukey’s test). | | | | | | | | | | | | | | | | | | | | | |
|  |  |  |  |  |  |  |  |  |  |  |  |  |  |  |  |  |  |  |  |  |  |
