# Supplementary material for: Astragaloside-IV prevents acute kidney injury and inflammation by normalizing muscular mitochondrial function associated with a nitric oxide protective mechanism in crush syndrome rats
Source: Ann Intensive Care. 2017 Sep 4;7:90. doi: 10.1186/s13613-017-0313-2 (PMC5583140; doi:10.1186/s13613-017-0313-2)
Supplement: Supplementary file 10 — Additional file 10: Table S7. Effect of fluid resuscitation on kidney function parameters by urine sample in CS rats. [file 13613_2017_313_MOESM10_ESM.docx]

| **SUPPLEMENTAL DIGITAL CONTENT Table 7. Effect of fluid resuscitation on kidney function parameters by urine sample in CS rats.** | | | | | | | | | | | | | | | | | | | | | |
| --- | --- | --- | --- | --- | --- | --- | --- | --- | --- | --- | --- | --- | --- | --- | --- | --- | --- | --- | --- | --- | --- |
|  |  |  |  |  |  |  |  |  |  |  |  |  |  |  |  |  |  |  |  |  |  |
|  |  | reperfusion (h) | | | | | | | | | | | | | | | | | | | |
|  |  | 0 | | |  | 1 | | |  | 3 | | |  | 6 | | |  | 24 | | |  |
| urine pH | sham | 6.0 | ± | 0.7 |  | 6.0 | ± | 0.7 |  | 6.0 | ± | 0.0 |  | 6.0 | ± | 0.0 |  | 6.3 | ± | 0.4 |  |
|  | CS only | 5.7 | ± | 0.4 |  | 5.3 | ± | 0.4 |  | 5.0 | ± | 0.0 | ^#^ | 5.0 | ± | 0.0 | ^#^ | 5.0 | ± | 0.0 | ^#^ |
|  | C-saline | 6.0 | ± | 0.0 |  | 5.7 | ± | 0.4 |  | 5.3 | ± | 0.4 |  | 5.0 | ± | 0.0 |  | 5.3 | ± | 0.4 |  |
|  | C-AS | 6.0 | ± | 0.0 |  | 6.3 | ± | 0.4 | ^*^ | 7.0 | ± | 0.0 | ^*^ | 6.7 | ± | 0.4 | ^*,†^ | 6.7 | ± | 0.4 | ^*,†^ |
| urine osmotic pressure | sham | 1.56 | ± | 0.05 |  | 1.65 | ± | 0.04 |  | 1.71 | ± | 0.13 |  | 1.84 | ± | 0.08 |  | 1.49 | ± | 0.09 |  |
|  | CS only | 1.95 | ± | 0.06 |  | 1.02 | ± | 0.32 |  | 0.60 | ± | 0.22 | ^#^ | 0.55 | ± | 0.15 | ^#^ | 0.38 | ± | 0.04 | ^#^ |
| (mOsm/kg・H_2_0) | C-saline | 1.76 | ± | 0.10 |  | 0.78 | ± | 0.03 |  | 0.94 | ± | 0.33 |  | 0.91 | ± | 0.14 | ^*^ | 0.73 | ± | 0.16 | ^*^ |
|  | C-AS | 1.80 | ± | 0.60 |  | 1.65 | ± | 0.01 | ^*,†^ | 1.65 | ± | 0.08 | ^*,†^ | 1.58 | ± | 0.03 | ^*,†^ | 1.00 | ± | 0.11 | ^*^ |
| urine volume | sham | 0.38 | ± | 0.02 |  | 0.42 | ± | 0.02 |  | 0.40 | ± | 0.05 |  | 0.35 | ± | 0.05 |  | 0.50 | ± | 0.06 |  |
|  | CS only | 0.41 | ± | 0.01 |  | 0.28 | ± | 0.01 | ^#^ | 0.22 | ± | 0.05 | ^#^ | 0.25 | ± | 0.04 | ^#^ | 0.27 | ± | 0.02 | ^#^ |
| (mL) | C-saline | 0.40 | ± | 0.25 |  | 0.54 | ± | 0.25 |  | 0.92 | ± | 0.04 | ^*^ | 0.40 | ± | 0.02 |  | 0.22 | ± | 0.02 |  |
|  | C-AS | 0.44 | ± | 0.30 |  | 0.40 | ± | 0.12 |  | 0.67 | ± | 0.02 | ^*^ | 0.45 | ± | 0.07 | ^*^ | 0.20 | ± | 0.07 |  |
| GFR | sham | 1.36 | ± | 0.25 |  | 1.89 | ± | 0.32 |  | 1.56 | ± | 0.12 |  | 1.95 | ± | 0.23 |  | 1.88 | ± | 0.25 |  |
|  | CS only | 1.23 | ± | 0.21 |  | 1.01 | ± | 0.45 |  | 0.90 | ± | 0.33 | ^#^ | 0.75 | ± | 0.57 | ^#^ | 0.78 | ± | 0.22 | ^#^ |
| (mL/min) | C-saline | 1.56 | ± | 0.46 |  | 1.98 | ± | 0.65 |  | 2.36 | ± | 0.15 | ^*^ | 1.65 | ± | 0.37 |  | 0.98 | ± | 0.08 |  |
|  | C-AS | 1.46 | ± | 0.33 |  | 1.79 | ± | 0.55 |  | 2.78 | ± | 0.33 | ^*^ | 2.00 | ± | 0.13 | ^*^ | 1.69 | ± | 0.68 | ^*^ |
| Values represent mean ± SEM (n = 3-6 each). ^#^P < 0.05 vs. sham group; ^*^P < 0.05 vs. CS-only group; ^†^P < 0.05 vs. C-saline group (Tukey’s test). | | | | | | | | | | | | | | | | | | | | | |
|  |  |  |  |  |  |  |  |  |  |  |  |  |  |  |  |  |  |  |  |  |  |
