# Supplementary material for: Astragaloside-IV prevents acute kidney injury and inflammation by normalizing muscular mitochondrial function associated with a nitric oxide protective mechanism in crush syndrome rats
Source: Ann Intensive Care. 2017 Sep 4;7:90. doi: 10.1186/s13613-017-0313-2 (PMC5583140; doi:10.1186/s13613-017-0313-2)
Supplement: Supplementary file 11 — Additional file 11: Table S8. Effect of fluid resuscitation on WBC and platelets in CS rats. [file 13613_2017_313_MOESM11_ESM.docx]

| **SUPPLEMENTAL DIGITAL CONTENT Table 8. Effect of fluid resuscitation on WBC and platelets in CS rats.** | | | | | | | | | | | | | | | | | | | | | |
| --- | --- | --- | --- | --- | --- | --- | --- | --- | --- | --- | --- | --- | --- | --- | --- | --- | --- | --- | --- | --- | --- |
|  |  |  |  |  |  |  |  |  |  |  |  |  |  |  |  |  |  |  |  |  |  |
|  |  | reperfusion (h) | | | | | | | | | | | | | | | | | | |  |
|  |  | 0 | | |  | 1 | | |  | 3 | | |  | 6 | | |  | 24 | | |  |
| WBC | sham | 47.4 | ± | 1.0 |  | 49.3 | ± | 0.4 |  | 47.3 | ± | 4.0 |  | 48.5 | ± | 6.4 |  | 47.5 | ± | 0.8 |  |
|  | CS only | 43.1 | ± | 4.0 |  | 48.0 | ± | 4.1 |  | 52.3 | ± | 8.8 |  | 58.8 | ± | 6.6 | ^#^ | 33.3 | ± | 0.2 | ^#^ |
| (× 10^2^/μL) | C-saline | 49.3 | ± | 3.2 |  | 48.3 | ± | 6.4 |  | 50.6 | ± | 7.2 |  | 55.3 | ± | 4.2 |  | 38.6 | ± | 0.9 |  |
|  | C-AS | 45.5 | ± | 5.3 |  | 40.5 | ± | 2.4 |  | 42.1 | ± | 4.5 |  | 44.5 | ± | 5.4 | ^*^ | 47.3 | ± | 0.3 | ^*,†^ |
| platelets | sham | 63.9 | ± | 3.1 |  | 67.3 | ± | 3.0 |  | 76.6 | ± | 6.0 |  | 71.7 | ± | 5.0 |  | 64.3 | ± | 8.6 |  |
|  | CS only | 70.1 | ± | 4.2 |  | 77.6 | ± | 1.1 |  | 75.3 | ± | 9.4 |  | 59.2 | ± | 5.1 | ^#^ | 33.8 | ± | 12.8 | ^#^ |
| (× 10^4^/μL) | C-saline | 68.3 | ± | 3.8 |  | 72.3 | ± | 6.3 |  | 70.3 | ± | 2.7 |  | 48.6 | ± | 3.8 |  | 40.1 | ± | 6.1 |  |
|  | C-AS | 77.0 | ± | 8.6 |  | 64.8 | ± | 11.7 |  | 63.7 | ± | 4.2 |  | 50.2 | ± | 3.7 | ^*^ | 61.3 | ± | 1.7 | ^*^ |
| Values represent mean ± SEM (n = 3-6 each). ^#^P < 0.05 vs. sham group; ^*^P < 0.05 vs. CS-only group; ^†^P < 0.05 vs. C-saline group (Tukey’s test). | | | | | | | | | | | | | | | | | | | | | |
|  |  |  |  |  |  |  |  |  |  |  |  |  |  |  |  |  |  |  |  |  |  |
