# Supplementary material for: Astragaloside-IV prevents acute kidney injury and inflammation by normalizing muscular mitochondrial function associated with a nitric oxide protective mechanism in crush syndrome rats
Source: Ann Intensive Care. 2017 Sep 4;7:90. doi: 10.1186/s13613-017-0313-2 (PMC5583140; doi:10.1186/s13613-017-0313-2)
Supplement: Supplementary file 12 — Additional file 12: Table S9. Effect of fluid resuscitation on RBC in CS rats. [file 13613_2017_313_MOESM12_ESM.docx]

| **SUPPLEMENTAL DIGITAL CONTENT Table 9. Effect of fluid resuscitation on RBC in CS rats.** | | | | | | | | | | | | | | | | | | | | | |
| --- | --- | --- | --- | --- | --- | --- | --- | --- | --- | --- | --- | --- | --- | --- | --- | --- | --- | --- | --- | --- | --- |
|  |  |  |  |  |  |  |  |  |  |  |  |  |  |  |  |  |  |  |  |  |  |
|  |  | reperfusion (h) | | | | | | | | | | | | | | | | | | | |
|  |  | 0 | | |  | 1 | | |  | 3 | | |  | 6 | | |  | 24 | | |  |
| RBC | sham | 732 | ± | 23 |  | 786 | ± | 96 |  | 756 | ± | 14 |  | 796 | ± | 38 |  | 704 | ± | 61 |  |
|  | CS only | 722 | ± | 87 |  | 887 | ± | 75 |  | 888 | ± | 29 |  | 1017 | ± | 78 | ^#^ | 1038 | ± | 104 | ^#^ |
| (× 10^4^/μL) | C-saline | 756 | ± | 24 |  | 780 | ± | 36 |  | 806 | ± | 56 |  | 900 | ± | 59 |  | 951 | ± | 13 |  |
|  | C-AS | 762 | ± | 65 |  | 750 | ± | 19 |  | 792 | ± | 25 |  | 887 | ± | 41 | ^*^ | 863 | ± | 79 | ^*^ |
| Values represent mean ± SEM (n = 3-6 each). ^#^P < 0.05 vs. sham group; ^*^P < 0.05 vs. CS-only group (Tukey’s test). | | | | | | | | | | | | | | | | | | | | | |
